# Complete genome sequences of *Providencia* bacteriophages PibeRecoleta, Stilesk and PatoteraRojo

**DOI:** 10.1186/s12863-023-01153-2

**Published:** 2023-09-01

**Authors:** Steven Batinovic, Hiu Tat Chan, Jason Stiles, Steve Petrovski

**Affiliations:** 1 Division of Materials Science and Chemical Engineering, Yokohama National University, Yokohama, Kanagawa Japan; 2 Department of Physiology, Anatomy, and Microbiology, La Trobe University, Bundoora, VIC Australia; 3 Department of Microbiology, Royal Melbourne Hospital, Parkville, VIC Australia

**Keywords:** Bacteriophage, *Providencia*, Genomics, Phage therapy

## Abstract

**Objectives:**

*Providencia* is a genus of gram-negative bacteria within the order *Enterobacterales*, closely related to *Proteus* and *Morganella*. While ubiquitous in the environment, some species of *Providencia*, such as *P. rettgeri and P. stuartii*, are considered emerging nosocomial pathogens and have been implicated in urinary tract infection, gastrointestinal illness, and travelers’ diarrhea. Given their intrinsic resistance to many commonly used antibiotics, this study aimed to isolate and sequence bacteriophages targeting a clinical *P. rettgeri* isolate.

**Data description:**

Here we report the complete genome sequence of three novel *Providencia* phages, PibeRecoleta, Stilesk and PatoteraRojo, which were isolated against a clinical *P. rettgeri* strain sourced from a patient in a metropolitan hospital in Victoria, Australia. The three phages contain dsDNA genomes between 60.7 and 60.9 kb in size and are predicted to encode between 72 and 73 proteins. These three new phages, which share high genomic similarity to two other *Providencia* phages previously isolated on *P. stuartii*, serve as important resources in our understanding about *Providencia* bacteriophages and the potential for future phage-based biotherapies.

## Objective

*Providencia* are a genus of gram-negative bacteria of the family *Morganellaceae*, closely related to *Proteus* and *Morganella*. There are nine currently recognized species within the genus, with *P. rettgeri, P. stuartii* and *P. alcalifaciens* the most encountered in the context of human disease. They are commonly associated with nosocomial urinary tract infection [1], particularly in patients with long-term urinary catheters leading to “purple bag syndrome” [2], associated with wounds in burn patients [3], and linked to diarrhea and gastroenteritis in children and travelers [4, 5]. Members of *Providencia* are intrinsically resistant to commonly used antibiotics including colistin, the last resort antibiotic for multi-resistant gram-negative bacteria [6]. Consequently, *Providencia* spp., along with other pathogenic bacteria of the order *Enterobacterales* including *Klebsiella pneumoniae*, *Escherichia coli*, *Enterobacter* spp., *Serratia* spp., *Proteus* spp. *and Morganella* spp. are classified as Critical Priority 1 on the World Health Organizations global priority pathogens list requiring further research and development of new antibiotics [7].

Bacteriophages (phages), viruses that are capable of infecting and killing bacteria, represent a potential solution to this emerging problem. Bacteriophages are ubiquitous in both natural and artificial environments and predicted to be the most abundant biological entities on the planet [8]. Only 26 *Providencia* phage genomes have thus far been deposited in the NCBI GenBank as of July 2023. Here we have isolated three novel *Providencia* phages, named PibeRecoleta, Stilesk and PatoteraRojo, by screening worm farm effluent samples obtained from various locations in Victoria, Australia on a clinical isolate of *P. rettgeri* (strain 9744). Visible phage plaques (approximately 0.3 mm) were picked, subjected to a total of three rounds of purification to ensure each plaque resulted from a single virion, and propagated as previously described [9].

## Data description

DNA were extracted from 1 ml phage filtrates (> 10^10^ PFU ml^− 1^) using a zinc chloride phenol:chloroform-based extraction [9, 10]. Isolated DNA (100 ng) were then prepared for sequencing using the NEBNext® Ultra™ II DNA Library Prep Kit (NEB) followed by sequencing on an Illumina MiSeq using a v3 600-cycle kit (Illumina) to generate 300 bp paired-end reads (n = 491,519–968,088 paired reads). Raw data were filtered using Trim Galore v0.6.4 with default settings (Q scores of ≥ 20, with automatic adapter detection) [11], and assembled with SPAdes v3.9.0 with default settings [12]. The assembled genome of PibeRecoleta was 60,727 bp with a GC content of 49.3% (1248-fold read coverage), Stilesk was 60,924 bp with a GC content of 49.5% (2310-fold read coverage) and PatoteraRojo was 60,728 bp with a GC content of 49.4% (1410-fold read coverage).

Genome termini were identified to be 11-bp 5’ cos overhangs (5’-GTGCGGAGAGC-3’) on all three phages using PhageTerm v1.0.12 [13] and confirmed by manual inspection of raw reads [10]. Genes were identified using Glimmer3 [14] followed by manual adjustment. Genomes were annotated using a combination of searching against the NCBI Conserved Domain Database [15] and the Virfam Webserver [16]. No tRNA genes were detected using tRNAscan-SE v2.0 [17] or Aragorn v1.2.41 [18]. All software was used with default parameters. A total of 72–73 predicted coding sequences were identified in each of the phage genomes. Those of which could be assigned a function (~ 30%) were characteristically organized in functional modules involved in virion morphogenesis and lysis, and DNA replication and nucleotide metabolism (Data file 1, Data file 2) [19].

The three highly syntenic phages described here share high pairwise DNA sequence similarity (78.7-89.7%) as determined using VIRIDIC (Data file 3) [19, 20]. Examination of public databases for sequenced phage genomes similar to these three phages revealed two highly related *Providencia* phages, Redjac [21] and PSTCR9, which were both isolated on *P. stuartii*. (Data file 3) [19]. Redjac and PSTCR9 phages, which represent the only two members of the *Redjacvirus* genus, share over 70% intergenomic similarity to the three *Providencia* phages sequenced here, indicating PibeRecoleta, Stilesk and PatoteraRojo phages belong to the *Redjacvirus* genus (Data file 3). Members of the *Redjacvirus* are also known to share moderate nucleotide similarity (46–47%) and similar genomic organization to phages from the well-studied flagellotropic *Chivirus* genus (such as *Enterobacteria* phage Chi) which target members of the *Enterobacteriaceae* (Data file 3) [19, 20, 22]. Insights into the molecular mechanisms utilized by these *Providencia* phages may potentially be gained from work already performed on *Chivirus* phages.

**Table 1:** Overview of data files/data sets

Label	Name of data file/data set	File types (file extension)	Data repository and identifier (DOI or accession number)
Data file 1	Table S1 - Phage genome annotations	MS Excel file (.xlsx)	figshare (10.6084/m9.figshare.23689797.v1) [19]
Data file 2	Figure S1 - Phage genome maps	PDF (.pdf)	figshare (10.6084/m9.figshare.23689797.v1) [19]
Data file 3	Figure S2 - Similarity of *Providencia* phages to publicly available phages	PDF (.pdf)	figshare (10.6084/m9.figshare.23689797.v1) [19]
Data set 1	Providencia phage vB-PreS-PibeRecoleta, complete genome	GenBank (.gbk)	NCBI nucleotide, https://identifiers.org/nucleotide:MT675124 [23]
Data set 2	Providencia phage vB-PreS-Stilesk, complete genome	GenBank (.gbk)	NCBI nucleotide, https://identifiers.org/nucleotide:MT675125 [24]
Data set 3	Providencia phage vB-PreS-PatoteraRojo, complete genome	GenBank (.gbk)	NCBI nucleotide, https://identifiers.org/nucleotide:MT675126 [25]
Data set 4	Raw reads of Providencia phages PibeRecoleta, Stilesk and PatoteraRojo	Raw sequence reads (.fastq)	NCBI BioProject, https://www.ncbi.nlm.nih.gov/bioproject?term=PRJNA1004027 [26]

## Limitations

*Providencia* phages PibeRecoleta, Stilesk and PatoteraRojo represent complete phage genomes. Our understanding of phages targeting *Providencia* is currently limited by the small sample size of phages isolated and described to date (n = 26). Isolation and description of additional *Providencia* phages in the future will facilitate increased knowledge of *Providencia* phage biology and their potential application as biotherapeutics.

## Data Availability

The data described in this Data note can be freely and openly accessed on NCBI GenBank under the following accession numbers: *Providencia* phage vB-PreS-PibeRecoleta, MT675124; *Providencia* phage vB-PreS-Stilesk, MT675125; *Providencia* phage vB-PreS-PatoteraRojo, MT675126. Raw sequence reads are available in the associated BioProject PRJNA1004027. Associated Data files are available on figshare (10.6084/m9.figshare.23689797.v1). Please see Table 1 and references [19, 23–26] for details and links to the data.

## Declarations

### Abbreviations

No abbreviations.
